# Human *Plasmodium knowlesi *infection in Ranong province, southwestern border of Thailand

**DOI:** 10.1186/1475-2875-11-36

**Published:** 2012-02-08

**Authors:** Natthawan Sermwittayawong, Balbir Singh, Mitsuaki Nishibuchi, Nongyao Sawangjaroen, Varaporn Vuddhakul

**Affiliations:** 1 Department of Microbiology, Faculty of Science, Prince of Songkla University, Hat Yai, Songkhla, Thailand; 2 Malaria Research Centre, Faculty of Medicine & Health Sciences, University Malaysia Sarawak, Kuching, Sarawak, Malaysia; 3 Center for Southeast Asian Studies, Kyoto University, Kyoto, Japan

**Keywords:** *Plasmodium knowlesi*, Thailand, Myanmar, Circumsporozoite protein

## Abstract

**Background:**

*Plasmodium knowlesi*, a simian malaria parasite, has been reported in humans in many Southeast Asian countries. In Thailand, most of the limited numbers of cases reported so far were from areas near neighbouring countries, including Myanmar.

**Methods:**

Blood samples collected from 171 Thai and 248 Myanmese patients attending a malaria clinic in Ranong province, Thailand, located near the Myanmar border were investigated for *P. knowlesi *using nested PCR assays. Positive samples were also investigated by PCR for *Plasmodium falciparum, Plasmodium vivax, Plasmodium malariae *and *Plasmodium ovale*, and were confirmed by sequencing the gene encoding the circumsporozoite protein (*csp*).

**Results:**

Two samples, one obtained from a Thai and the other a Myanmese, were positive for *P. knowlesi *only. Nucleotide sequences of the *csp *gene derived from these two patients were identical and phylogenetically indistinguishable from other *P. knowlesi *sequences derived from monkeys and humans. Both patients worked in Koh Song, located in the Kawthoung district of Myanmar, which borders Thailand.

**Conclusion:**

This study indicates that transmission of *P. knowlesi *is occurring in the Ranong province of Thailand or the Kawthoung district of Myanmar. Further studies are required to assess the incidence of knowlesi malaria and whether macaques in these areas are the source of the infections.

## Background

There are more than 200 species of *Plasmodium *that infect a variety of hosts, including reptiles, birds, rodents, primates and other mammals [1]. However, only four species (*Plasmodium falciparum*, *Plasmodium vivax*, *Plasmodium malariae *and *Plasmodium ovale*) are well-known infectious agents that cause malaria in humans. Recently, humans infected with *Plasmodium knowlesi*, a simian malaria parasite [1], have been described in a number of Southeast Asian countries including Malaysia [2,3], Singapore [4], Myanmar [5], Vietnam [6], Indonesia [7], the Philippines [8] and Thailand [9]. Under the microscope, the early trophozoites of *P. knowlesi *cannot be distinguished from those of *P. falciparum *and the other blood stages are identical to *P. malariae*. Therefore, molecular detection methods are required for the accurate identification of *P. knowlesi*.

**Figure 1:**
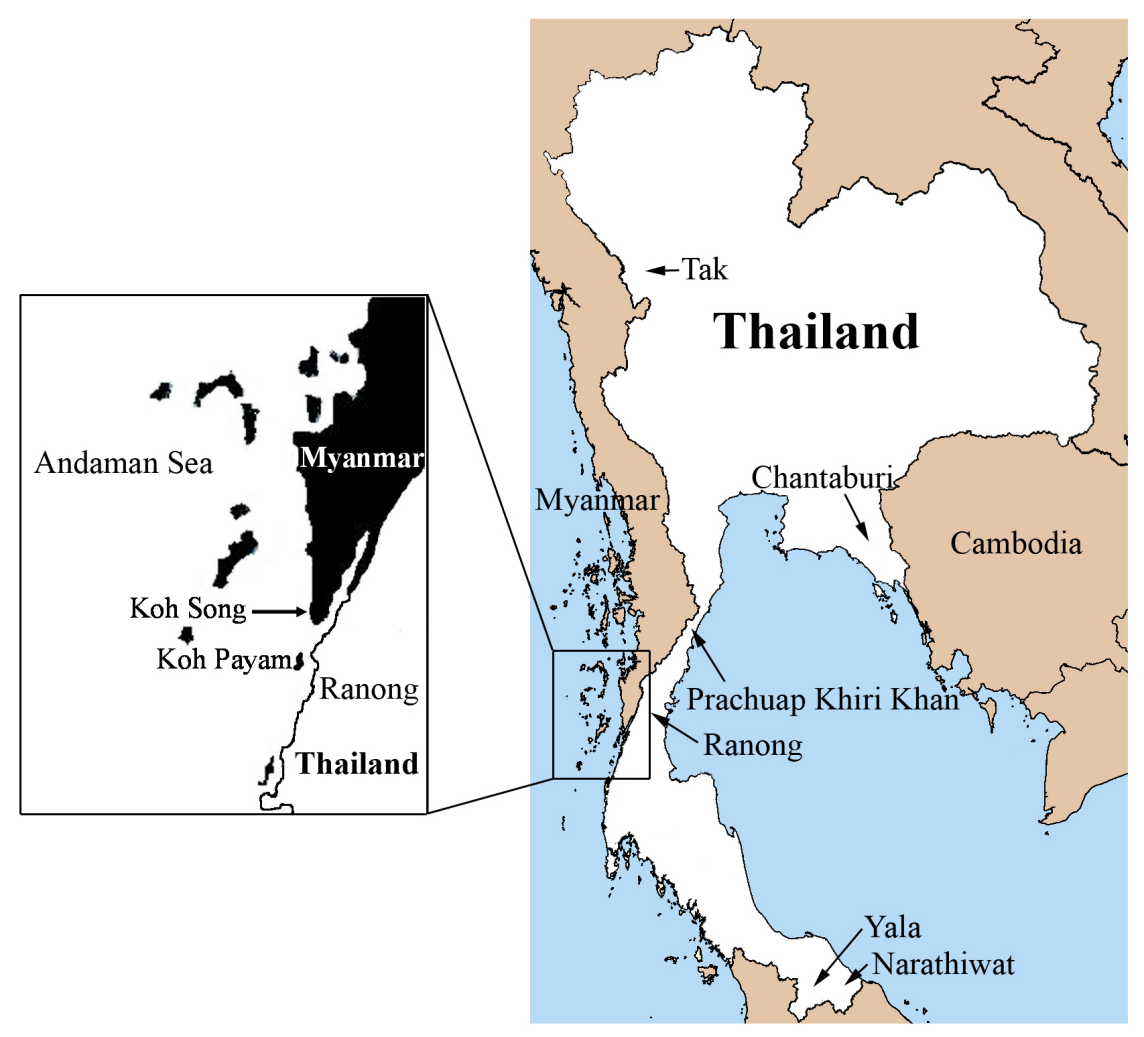
**Map of Ranong province close to Koh Song and Koh Payam**.

In Thailand, the first case of a human *P. knowlesi *infection was acquired in Prachuap Khiri Khan, a southern Thai province, and reported in 2004 [9]. No other humans infected with this species of *Plasmodium *were reported in Thailand until 2009, when 10 cases from Tak, Prachuap Khiri Khan, Chantaburi, Yala and Narathiwat provinces were described [10]. These areas located near borders of Myanmar, Cambodia, and Malaysia (Figure 1). Recently, a total of 23 *P. knowlesi *infected patients have been reported in these border areas [11]. Retrospective study of blood samples obtained from malaria patients living in Tak province in 1996 indicated that *P. knowlesi *has circulated among humans in Thailand more than a decade [11]. Ranong is one of Thailand's southern provinces located on the coast of the Andaman Sea. The province shares a border with Myanmar (Figure 1). There is extensive trading and labour flow between Myanmese and the Thais, with Myanmese frequently crossing the border into Thailand and some seeking health treatment in Ranong. In order to assess whether there were additional cases of human knowlesi malaria, particularly among the Myanmese, blood samples collected from malaria patients attending the malaria clinic at the Vector Borne Disease Control Center at Ranong were investigated.

## Methods

### Sample collection and malarial DNA extraction

This study was approved by the Ethics Committee of Prince of Songkla University, Thailand. Four hundred and nineteen blood samples were collected from 171 Thai and 248 Myanmese patients attending a malaria clinic in Ranong province, Thailand from May 2008 to June 2010. Blood films prepared from finger-prick blood samples were stained with Giemsa and malarial species was identified by microscopic examination. Concomitantly, filter paper (903 Protein Saver Card; Whatman Ltd., NJ, USA) was used to absorb a drop of blood from each patient. DNA was extracted using a DNA extraction kit (QIAGEN, Germany) and this served as the DNA template for nested PCR assays.

### *Plasmodium knowlesi *identification by Nested PCR

First round PCR amplification of the small subunit of ribosomal RNA (SSU rRNA) genes was performed using the Plasmodium genus-specific oligonucleotide primers rPLU1 (5'-TCAAAGATTAAGCCATGCAAGTGA-3') and rPLU5 (5'-CCTGTTGTTGCCTTAAACTCC-3') [12]. A plasmid containing a *P. knowlesi *SSU rRNA insert from a patient with knowlesi malaria served as the positive control. PCR was conducted in a 20 μL reaction mixture containing 1x PCR buffer, 2 mM MgCl_2_, 0.2 mM of each dNTP, 0.25 mM of each primer, 1 unit of *Taq *DNA polymerase (Promega), and 2 mL of DNA template. The PCR conditions were as follows: 94°C for 4 min, 35 cycles at 94°C for 30 sec, 55°C for 1 min, 72°C for 1 min, followed by a final extension for 4 min at 72°C. Two microlitres of the first round PCR amplification product was used as DNA template for the second round PCR to confirm the presence of the *P. knowlesi *species using the primers Pmk8 (5'-GTTAGCGAGAGCCACAAAAAAGCGA-3') and Pmkr9 (5'-ACTCAAAGTAACAAAATCTTCCGTA-3') [3]. The PCR conditions were the same as described in the first round except that the annealing temperature was 58°C [3]. PCR products were analysed by electrophoresis on 1.5% agarose gel and detected by staining with ethidium bromide.

### Amplification and analysis of *Plasmodium knowlesi *circumsporozoite protein gene

In order to confirm infection by *P. knowlesi*, the *Plasmodium *circumsporozoite protein (*csp*) gene of the two positive samples was amplified by nested PCR using PkCSP-F (5'-TCCTCCACATACTTATATACAAGA-3') and PkCSP-R (5'-GTACCGTGGGGGACGCCG-3') primers [3], for the first round and the N2F-PkCSP (5'-CGGGATCCCCACACACTTCGAA-3') and N2R-PkCSP (5'-AACTGCAGCCATTACACAAGCTTCCAC-3') primers for the second round PCR. The PCR products were purified using QIAquick Gel Extraction Kit (QIAGEN, Germany) and subjected to sequencing.

### Analysis of DNA sequences

Sequence analysis was performed with *csp *gene sequences of the non-repeat region of the circumsporozoite proteins from *Plasmodium *spp. as described previously [3] using the ClustalW program. Phylogenetic and molecular evolutionary analyses were conducted using MEGA version 3.1 [13]. The unrooted phylogenetic tree was constructed by the neighbour-joining method with the bootstrap re-sampling of 1,000 replicates.

## Results

### Microscopy results

By microscopy, 69 and 102 Thai blood samples were identified as *P. falciparum *and *P. vivax*, respectively whereas 121, 123 and four Myanmese blood samples were identified as *P. falciparum, P. vivax *and a mixed infection (*P. falciparum *and *P. vivax*), respectively.

### PCR analysis for *Plasmodium knowlesi*

**Figure 2:**
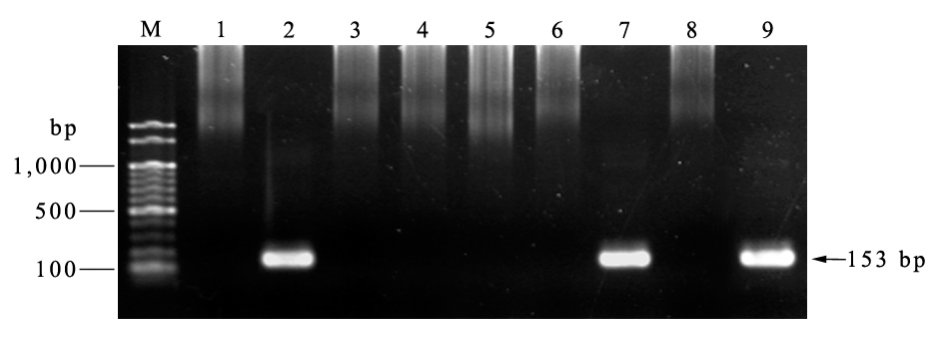
**Nested PCR screening of blood samples collected for *Plasmodium knowlesi *Lanes M = Molecular weight marker (100 bp ladder -New England Biolabs); lane 1 = negaitve control, lane 2 = positive control; lane 3-6 and 8 = negative blood samples; lane 7 and 9 = positive blood samples (M2/20 and M2/51 respectively)**.

A total of 419 blood samples were investigated and a 153-bp SSU rRNA fragment specific for *P. knowlesi *was detected in only two samples (designated as M2/20 and M2/51) (Figure 2). One sample had been obtained from a 45 year-old Thai male and the other was from a 30 year-old Myanmese male. Both samples were collected in the same period of time (June 2008) and were diagnosed by microscopy as *P. vivax *infections. These samples were examined for co-infection with other *Plasmodium *spp. by nested PCR using species-specific primers for *P. falciparum*, *P. malariae*, *P. ovale *and *P. vivax *[12]. Both samples were negative, confirming that they were each a single infection of *P. knowlesi*.

### Analysis of the circumsporozoite protein gene

**Figure 3:**
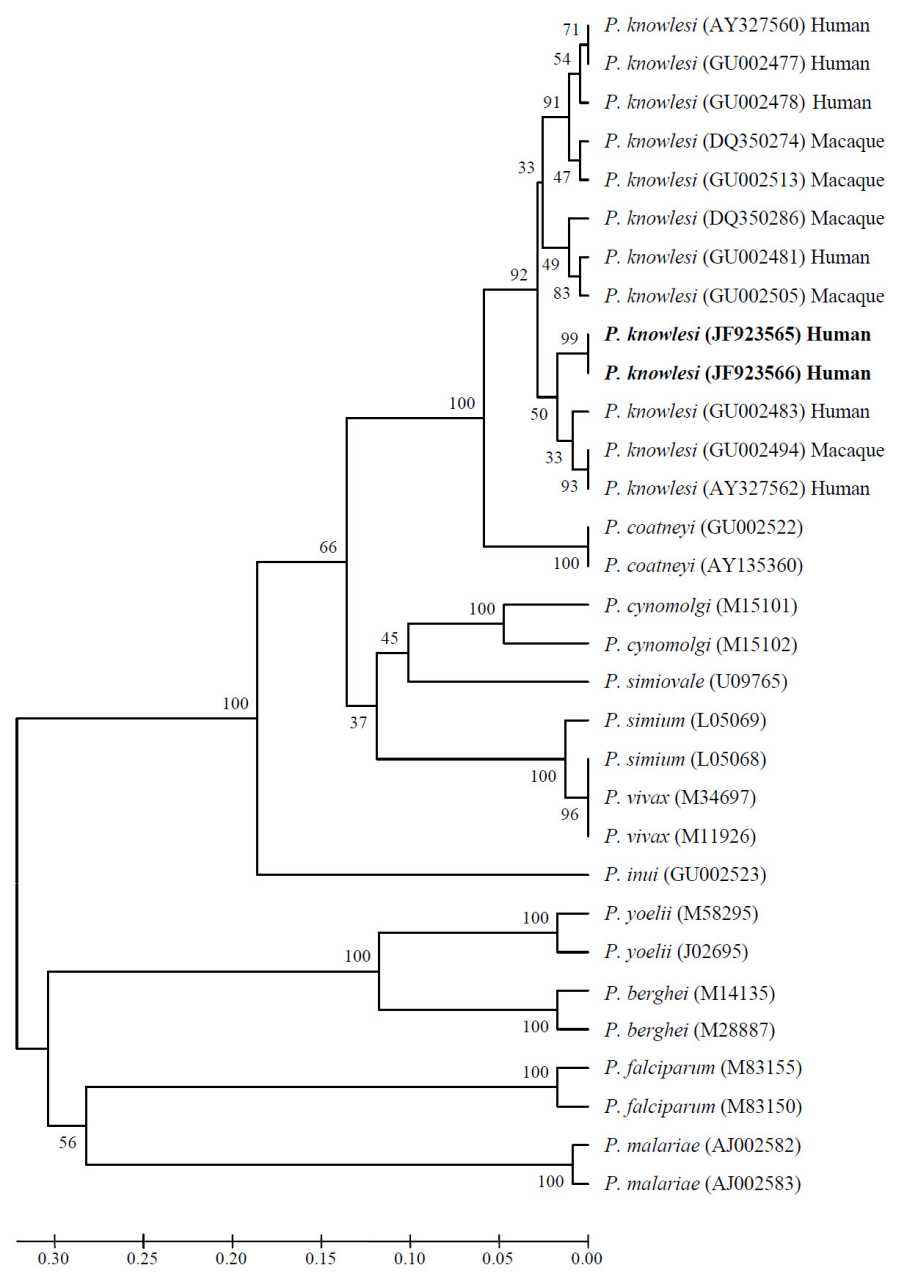
**Phylogenetic tree based on the non-repeat region of the circumsporozoite protein gene**. The *P. knowlesi *sequences in bold are from the current study. Numbers on the branches are bootstrap percentages based on 1,000 replicates. GenBank accession numbers are indicated in the brackets.

The nucleotide sequences of the *csp *gene of *P. knowlesi *obtained from the Thai (M2/20) and Myanmese (M2/51) patient were identical [GenBank accession numbers: JF923565 and JF923566]. Phylogenetic analysis of the non-repeat region indicated that these sequences were indistinguishable from those of *P. knowlesi *derived from monkey and human infections in Malaysia and Thailand (Figure 3), thereby confirming that the two persons were infected with *P. knowlesi*.

## Discussion

In Myanmar, a patient infected with *P. knowlesi *was first detected in Northern Myanmar [14]. This patient was a camp worker. In 2008, investigation of 146 blood samples collected in southern Myanmar near Yunnan province of China revealed that four samples were positive for *P. knowlesi *and that 28 samples showed co-infection of *P. knowlesi *with *P. falciparum*, *P. vivax*, or both [5]. In Thailand, the first case of *P. knowlesi *infection was reported in 2004, of a patient who lived in Bangkok, Thailand and had recently visited the southern province of Prachuap Khiri Khan near the Myammar border [9]. In addition, 10 and 23 more *P. knowlesi *infections have been identified in 2006-2007 and 2008-2009, respectively in Tak, Chantaburi, Prachuap Khiri Khan, Yala and Narathiwat provinces [10,11]. Interestingly, all of these provinces are located near the Myanmar border (Tak and Prachuap Khiri Khan), Cambodia border (Chantaburi), and Malaysia border (Yala and Narathiwat). In this study, two cases of *P. knowlesi *infection detected at the Vector Borne Disease Control Center in Ranong province, which is close to the Myanmar border are reported. One patient was a Thai who worked in a rubber plantation in Koh Song, in the Kawthoung District of Myanmar and the other was Myanmese who worked as a fisherman in the same area. Koh Song is close to Ranong province (Figure 1), therefore, transmission of this malaria parasite species near this Thai border is suspected. Recently, there has been a report that a French tourist was infected with *P. knowlesi *at Koh Payam (Figure 1) near Ranong province [15]. Wild macaques presence in the forest fringe near this tourist's bungalow were expected to be reservoirs of *P. knowlesi*. It is possible that human carriers of *P. knowlesi *exist in this and surrounding areas and monkey-to-human transmission of *P. knowlesi *may be taking place. Most primats observed in Thailand are long-tailed macaques (*Macaca fascicularis*) [16]. Increasing in natural forest invasion disturbs habitats of these macaques and has caused them to migrate to forest fringes, temples or parks in urban areas near human settlements. In addition, some macaques are considered as pets to humans [17]. Malaria among long-tailed macaques in southern Thailand was evaluated in Ranong and Prachuap Khiri Khan provinces by amplification and sequencing of the SSU rRNA and the mitochondria cytochrome *b *genes. Non-human primate malaria (*P. inui *and *P. coatneyi*) and *Hepatocystis *species were detected only in wild macaques in Ranong mangrove forests where anopheline mosquitoes were abundant [17]. However, *P. knowlesi *was not detected in these macaques. Nevertheless, transmission of *P. knowlesi *from monkey to human cannot be ruled out.

It is considered important to correctly identify the malaria species and clarify the mode of transmission for establishing appropriate preventive measures. In Thailand, *P. falciparum *and *P. vivax *are most frequently detected in patients [10]. In this study, malaria parasites in blood samples from a Thai and a Myanmese patient were probably in the late erythrocyte stage, so they were originally identified as *P. vivax *instead of *P. malariae *by microscopy. Thus, health care workers in this area who are responsible for identification of malaria species should be trained appropriately so that malaria species including *P. knowlesi *can be correctly identified.

The nucleotide sequences of the *csp *gene of *P. knowlesi *obtained from the two patients were identical. Comparison of these two *P. knowlesi csp *gene sequences and the other *Plasmodium csp *gene sequences deposited in GenBank confirmed that the *P. knowlesi *sequences obtained from the Thai and Myanmese patients were closely related to *P. knowlesi *derived from both monkeys and humans (Figure 3). Blood from these patients was collected in the same month (June 2008) and it is possible that they may share the same origin. More in-depth epidemiological studies, including entomological investigations should be undertaken around Ranong and surrounding areas to determine the incidence of *P. knowlesi *and to understand the mode of transmission of this malaria parasite in the Thai-Myanmar border areas.

## Conclusion

Two blood samples, one obtained from a Thai and from a Myanmese, were each single infections of *P. knowlesi*. Both of them work in the Kawthoung district of Myanmar that is close to Ranong province, Thailand and they visited Ranong to seek treatment. This indicates that this Thai-Myanmar border area is one of areas where *P. knowlesi *is being transmitted.

## Competing interests

The authors declare that they have no competing interests.

## Authors' contributions

NK carried out the PCR analysis and wrote the manuscript. BS provided *P. knowlesi *positive control and helped in manuscript preparation. NS organized blood collection and helped in PCR analysis. MN provided some research funds and helped in study design. VV helped in writing the paper and discussion. All authors read and approved the submitted manuscript.
